# A novel data mining application to detect safety signals for newly approved medications in routine care of patients with diabetes

**DOI:** 10.1002/edm2.237

**Published:** 2021-04-06

**Authors:** Michael Fralick, Martin Kulldorff, Donald Redelmeier, Shirley V. Wang, Seanna Vine, Sebastian Schneeweiss, Elisabetta Patorno

**Affiliations:** ^1^ Division of Pharmacoepidemiology and Pharmacoeconomics Department of Medicine Brigham and Women's Hospital and Harvard Medical School Boston MA USA; ^2^ Sinai Health System and the Department of Medicine University of Toronto Toronto ON Canada; ^3^ Sunnybrook Research Institute Sunnybrook Health Sciences Centre Toronto ON Canada; ^4^ ICES Sunnybrook Health Sciences Centre Toronto ON Canada

**Keywords:** data mining, diabetes, medication

## Abstract

**Background:**

Clinical trials are often underpowered to detect serious but rare adverse events of a new medication. We applied a novel data mining tool to detect potential adverse events of canagliflozin, the first sodium glucose co‐transporter 2 (SGLT2 inhibitor) in the United States, using real‐world data from shortly after its market entry and before public awareness of its potential safety concerns.

**Methods:**

In a U. S. commercial claims dataset (29 March 2013–30 Sept 2015), two pairwise cohorts of patients over 18 years of age with type 2 diabetes (T2D) who were newly dispensed canagliflozin or an active comparator, that is a dipeptidyl peptidase 4 inhibitor (DPP4) or a glucagon‐like peptide 1 receptor agonist (GLP1), were identified and propensity score‐matched. We used variable ratio matching with up to four people receiving a DPP4 or GLP1 for each person receiving canagliflozin. We identified potential safety signals using a hierarchical tree‐based scan statistic data mining method with the hierarchical outcome tree constructed based on international classification of disease coding. We screened for incident adverse events where there were more outcomes observed among canagliflozin vs. comparator initiators than expected by chance, after adjusting for multiple testing.

**Results:**

We identified two pairwise propensity score variable ratio matched cohorts of 44,733 canagliflozin vs. 99,458 DPP4 initiators, and 55,974 canagliflozin vs. 74,727 GLP1 initiators. When we screened inpatient and emergency room diagnoses, diabetic ketoacidosis was the only severe adverse event associated with canagliflozin initiation with *p* < .05 in both cohorts. When outpatient diagnoses were also considered, signals for female and male genital infections emerged in both cohorts (*p* < .05).

**Conclusions and relevance:**

In a large population‐based study, we identified known but no other adverse events associated with canagliflozin, providing reassurance on its safety among adult patients with T2D and suggesting the tree‐based scan statistic method is a useful post‐marketing safety monitoring tool for newly approved medications.

## Graphical abstract

Clinical trials are often underpowered to detect serious but rare adverse events of a new medication. We applied a novel data mining tool to detect potential adverse events of canagliflozin, the first SGLT2 inhibitor in the United States, using real‐world data from shortly after its market entry and before public awareness of its potential safety concerns. In a large population‐based study, we identified known but no other adverse events associated with canagliflozin, providing reassurance on its safety among adult patients with T2D and suggesting the tree‐based scan statistic is a useful post‐marketing safety monitoring tool for newly approved medications.

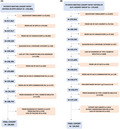

## 1 INTRODUCTION

Identifying adverse events of a newly approved medication is initially based on the results of clinical trials.^1^, ^2^, ^3^, ^4^ This can be problematic since medications are typically approved based on 1 or 2 pivotal clinical trials that may enrol less than 1000 patients per drug and often select healthier patients than in usual care.^5^, ^6^ While the pre‐approval trials may provide information on common adverse events, rare serious adverse events may go undetected.^7^, ^8^ Until additional safety data are actively reported (eg voluntary reports regulators) or published in the scientific literature (eg observational studies), clinicians rely on relatively scarce data to evaluate a medication's safety.

Detecting drug‐related adverse events, in particular rare events, generally requires a large sample size and prior knowledge of the potential association with a specific adverse event.^7^, ^9^ However, prior knowledge is often limited when a drug first enters the market. To detect unsuspected adverse reactions, data mining tools are advantageous as they are hypothesis‐free^10^, ^11^ and can leverage information for millions of patients and thousands of potential outcomes when used in the context of longitudinal data sources such as healthcare claims data.^12^, ^13^

Tree‐based scan statistics are a data mining approach implemented by the free TreeScan™ software (www.treescan.org), which can evaluate a wide range of health outcomes, arranged in a hierarchical tree, while adjusting for multiple testing.^10^, ^11^, ^14^, ^15^, ^16^ In pharmacovigilance, TreeScan was initially used to evaluate vaccine safety and was recently implemented by the Food and Drug Administration (FDA) to monitor the short‐term safety of the human papillomavirus vaccine.^10^, ^14^, ^15^ TreeScan has also been used to determine if it can identify well‐established side effects of widely used medications, including diabetes medications and antifungal medications that have been in use for decades.^10^ However, whether a more recently proposed method that combines TreeScan with propensity score‐matched analysis in the context of a new‐user active comparator study design can be used to reliably identify drug‐related adverse events among patients with diabetes using newly approved medications remains unknown.^17^ Thus, we sought to evaluate whether TreeScan combined with propensity score matching and a new‐user active comparator design could help identify incident adverse events of a newly approved diabetes medication shortly after its market entry. This was implemented in a cohort study of adult patients with type 2 diabetes (T2D) initiating canagliflozin, the first marketed sodium glucose co‐transporter 2 (SGLT2) inhibitors in the United States, compared to two active comparators between March 2013 and September 2015.

## 2 METHODS

### 2.1 Study population

We conducted a population‐based, new‐user, cohort study using data from the IBM MarketScan database.^12^ This database includes patient demographics and longitudinal, patient‐level data on healthcare utilization, inpatient and outpatient diagnostic tests and procedures, and pharmacy dispensing of drugs to over 50 million patients in the United States.^12^

**Figure 1:**
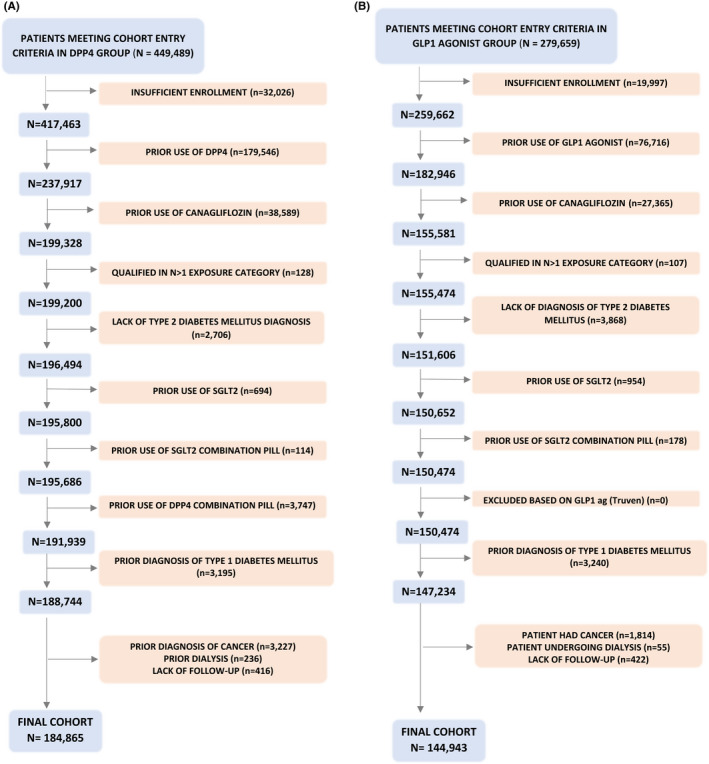
Cohort entry criteria and flow diagram of the two study cohorts

We compared adults with T2D who were newly prescribed canagliflozin or one of two comparators: a dipeptidyl peptidase 4 (DPP4) inhibitor (ie sitagliptin, saxagliptin, linagliptin, alogliptin) or a glucagon‐like peptide 1 (GLP1) receptor agonist (ie exenatide, liraglutide, albiglutide, dulaglutide) in two pairwise comparisons between 29 March 2013 (date of approval of canagliflozin in the United States) and 30 September 2015 (last available data) (Figure 1). We focused on canagliflozin because it made up more than 90% of SGLT2 prescribing during this time period. Patients with diabetes mellitus type 2 were identified using the *International Classification of Diseases*, *Ninth Revision* (ICD‐9) codes similar to previous studies.^18^ New users of canagliflozin or a DPP4 inhibitor were defined as those without a prior prescription for an SGLT2 inhibitor or a DPP4 inhibitor in the preceding 180 days. Similarly, new users of canagliflozin or a GLP1 agonist were defined as those without a prior prescription for an SGLT2 inhibitor or a GLP1 agonist in the preceding 180 days. Cohort entry date was the date of first prescription. DPP4 inhibitors and GLP1 agonists were chosen as the comparator medications because during the study period they were considered as a second‐line treatment for diabetes, similar to SGLT2 inhibitors.^7^

Patients receiving both canagliflozin and a comparator on the cohort entry date were excluded. Patients with any of the following characteristics in the 180 days prior to cohort entry were also excluded: insufficient enrolment (ie less than 180 days of baseline data), end‐stage renal disease or cancer. The latter two were identified using ICD9 codes similar to prior studies.^18^ The Brigham and Women's Hospital Institutional Review Board provided ethics approval and a valid data use agreement for the IBM MarketScan (‘MarketScan’) database was in place.

### 2.2 Cohort follow‐up

Follow‐up began on the day after cohort entry and continued until the first occurrence of the end of the study period (ie the first of: 30 September 2015, 180 days after the index date, end of continuous health plan enrolment, discontinuation of the initial medication or switching to or adding one of the comparator medications, or death). The follow‐up period was truncated at 180 days since we were interested in acute rather than long‐term adverse reactions.^10^ A medication was considered discontinued if 60 days elapsed after the expiration of the last prescription's supply.^7^, ^18^

### 2.3 Baseline covariates

Patient demographics and characteristics were assessed during the 180 days before cohort entry. The characteristics were selected based on diagnoses and procedures covered: chronic medical conditions, markers of diabetes severity, healthcare utilization, diabetes medications and non‐diabetes‐related medications.

### 2.4 Hierarchical tree of potential outcomes

The potential outcomes to be included in our hierarchical classification system (‘tree’) were developed using ICD‐9 diagnosis codes. We removed outcomes that were unlikely to represent an acute drug‐related adverse event: ICD9 140 – 239 (neoplasms), ICD9 630 – 679 (pregnancy), ICD9 740 – 759 (congenital), but included all other ICD9 codes. There are five levels to ICD9 diagnosis codes. Level 1 is the broadest category and spans entire disease categories (eg ICD9 codes 001–139 [Infectious and parasitic diseases]). Level 2 includes subgroups of disease or injury ICD9 categories (eg ICD9 codes 130–136 [other infections and parasitic diseases]). Level 3 includes individual ICD9 codes without a decimal value (eg 010 [primary tuberculosis infection]), and level 4 generally includes ICD9 codes with one decimal value (eg 010.0 [primary tuberculosis complex]), while level 5 generally includes ICD9 codes with more than one decimal value (eg 010.00 [Primary tuberculous infection, unspecified]). The increasing level of specificity from level 1 to level 5 creates a hierarchical tree structure.

### 2.5 Incident outcomes

We defined an incident outcome as the first inpatient or emergency department diagnosis code that occurred during a patient's available follow‐up time for which there was not another inpatient, emergency department or outpatient diagnosis with the same ICD‐9 code during the 180 day period.^10^ Specifically, in the tree looking at second level outcomes, if the exact second level outcome occurred in the preceding 180 days (in addition to the 180 days before the index date), then this event would not be counted. This step was purposeful to increase the likelihood of identifying real incident events, rather than pre‐existing chronic medical conditions. If there were more than one potential incident outcome on the same day, we selected the one that was less common based on the frequency of the code in our dataset.^10^ This approach is in line with prior studies applying TreeScan because a key aim of this approach is to detect rare adverse events and has the goal to reduce the likelihood of false signals.

Incident outcomes were assessed at the second, third, fourth and fifth level of the ICD9 hierarchical tree. Potential outcomes at level 1 of the tree were not considered because of the broad nature of these categories. In secondary analyses, we also explored incident outcomes based on outpatient diagnoses in addition to inpatient and emergency visits. This was to assess signals for potential adverse events that may be managed in an outpatient setting without requiring a hospitalization or an emergency department visit.

### 2.6 Statistical analysis

Propensity score (PS) matching methodology was used to adjust for confounding using a nearest neighbour matching within a caliper of 0.05.^17^ The probability of initiating canagliflozin versus a DPP4 inhibitor or a GLP1 agonist was calculated through a multivariable logistic regression model which contained all of the potential confounders at baseline. The estimated PS was used to match initiators of canagliflozin with initiators of a comparator, using variable ratio matching with up to 4 comparators to each canagliflozin initiator. Covariate balance between the matched cohorts was assessed using standardized differences.^19^ A standardized difference of 0.1 or less indicates negligible differences between groups.^19^ The standardized differences were calculated for each of the two pairwise comparisons.

The TreeScan method tests the null hypothesis of no difference in risk of adverse events in any outcome node in the tree against a one‐sided alternative that there is at least one outcome node where the risk of adverse events is higher in the exposed group than in the comparator group. When screening potential multiple outcomes for signal identification, it is critical to control the rate of false positives. TreeScan generates multiplicity‐adjusted *p*‐values that accurately reflect the type I error rate in the absence of confounding.^10^, ^16^, ^17^, ^20^, ^21^, ^22^, ^23^ That is, if there is not a single outcome with an excess risk, we have a 95% probability of finding zero signals.

**Figure A1:**
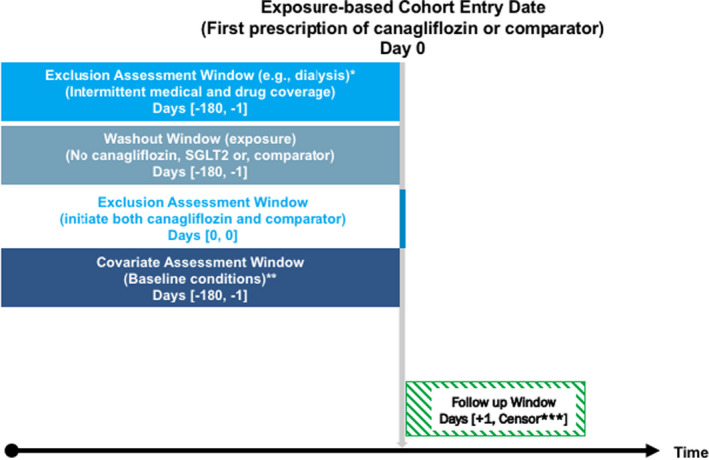
Study design

**Figure A2:**
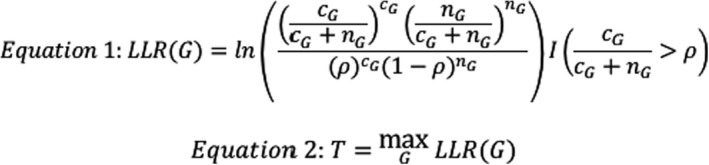
Formula for calculating the log likelihood ratio. LLR = log likelihood ratio, G = node of interest, ln = natural log, T = unconditional Bernoulli tree scan statistic, c_G_ = number of cases in the exposure group for a given node G, n_G_ = number of cases in the comparator group for a given node G, *p* = probability of being in the exposure group (for 1:1 matched, this is 0.5)

We used the unconditional Bernoulli tree‐based scan statistic. To meet the assumptions of this statistic, all patients within each matched set were censored at the end of follow‐up of the canagliflozin initiator or at the end of follow‐up of the uncensored comparator initiator with the longest follow‐up, whichever came first. Failing to do so would result in differential follow‐up time making it challenging to know if an observed signal is related to a true adverse event or is instead related to a longer follow‐up period for detection. The log‐likelihood ratio for each node was calculated based on the number of cases in the exposed (ie canagliflozin) or comparator group (ie DPP4 inhibitors or GLP1 agonists) as well as the probability of being in the exposed group (Appendix Figure A1). For our matched cohort, this probability was set to the proportion of patients receiving canagliflozin or a comparator. Since the distribution of the tree‐based scan statistic method is unknown, we derived multiple testing adjusted *p*‐values non‐parametrically using Monte Carlo hypothesis testing where permutations of the data are generated under the null hypothesis.^10^ The multiple testing adjusted *p*‐value was determined by ranking the test statistics from 9,999 datasets simulated under the null and the observed dataset from largest to smallest. The *p*‐value was calculated as the rank of the observed dataset test statistic divided by 10,000 (9,999 simulated datasets +1 observed dataset). The multiplicity‐adjusted *p*‐values were interpreted as the probability of seeing an association of the observed magnitude or one more extreme if the null hypothesis was true. Together with the relative risk estimates, these *p*‐values were used as a means to prioritize alerts for further investigation (Appendix Figure A2). Specifically, we rank ordered the signals by their *p*‐value from lowest to highest *p*‐value. As a surveillance method to detect potential problems, the alerts should not determine whether there is an association without such a follow‐up investigation. Rate ratios and rate differences per 1,000 person years were calculated nominally.

The cohort was generated using R version 3.4.2 in the validated Aetion platform.^24^ The hierarchical tree was built using SAS Version 9.4 and scanned using the free TreeScan v9.4 software available at: www.treescan.org.

## 3 RESULTS

### 3.1 Study population

After the application of the study selection criteria (Figure 1), we identified 44,733 PS matched patients who were newly prescribed canagliflozin and 99,458 PS matched patients who were newly prescribed a DPP4 inhibitor in the canagliflozin vs. DPP4 inhibitors pairwise cohort, and 55,974 canagliflozin initiators and 74,727 GLP1 agonist initiators in the canagliflozin vs. GLP1 agonists pairwise cohort. Thus, over 75% of the people newly prescribed canagliflozin were matched to people newly prescribed a GLP1 agonist or DPP4 inhibitor (Appendix Table A1). All differences in patient characteristics were well balanced, as assessed by standardized differences. Across the two pairwise cohorts, study participants had average age of 55 years, 9% had history of ischaemic heart disease, and 4% had a recent hospitalization. Patients included in the canagliflozin vs. DPP4 inhibitor pairwise cohort were more frequently males compared with patients included in the canagliflozin vs. GLP1 agonists pairwise cohort (53% vs. 50%), and they were more frequently treated with metformin (63% vs. 56%), less frequently treated with insulin (23% vs. 26%) and had less frequent visits with an endocrinologist (11% vs. 15%). (Table 1). The average duration of follow‐up was approximately 19 weeks.

**Table 1:** Baseline patient characteristics of canagliflozin initiators vs. initiators of other diabetes drugs in two pairwise propensity score‐matched cohorts

Patient characteristics^a^	Canagliflzoin vs. DPP4i			Canagliflozin vs. GLP−1RA		
	Canagliflozin (*n *= 44,733)	DPP4i^b^ (*n *= 99,458)	St. diff	Canagliflozin (*n *= 55,974)	GLP−1RA^b^ (*n *= 74,727)	St. diff
Age (years), mean (SD)	54.9 (9.6)	54.7 (10.9)	0.02	54.7 (9.9)	54.7 (10.0)	0.00
Male, %	23,689 (53.0)	23,698 (53.0)	0.00	27,963 (50.0)	27,901 (49.8)	0.00
Diabetes severity						
Diabetic nephropathy, %	1,666 (3.7)	1,654 (3.7)	0.00	2,276 (4.1)	2,319 (4.1)	0.00
Diabetic retinopathy, %	1,822 (4.1)	1,812 (4.1)	0.00	2,336 (4.2)	2,334 (4.2)	0.00
Diabetic neuropathy, %	3,872 (8.7)	3,862 (8.6)	0.00	5,281 (9.4)	5,303 (9.5)	0.00
Number of diabetes medications, mean (SD)	1.1 (0.9)	1.1 (0.8)	0.00	1.2 (0.9)	1.1 (0.9)	0.00
Metformin, %	27,952 (62.5)	28,148 (62.9)	−0.01	31,655 (56.6)	31,541 (56.4)	0.00
Insulin, %	10,356 (23.2)	10,054 (22.5)	0.02	14,700 (26.3)	14,805 (26.5)	0.00
GLP1 agonists, %	3,649 (8.2)	3,262 (7.3)	0.03	–	–	–
DPP4 inhibitors, %	–	–	–	9,388 (16.8)	9,293 (16.6)	0.00
Other conditions						
Hypertension, %	28,037 (62.7)	27,935 (62.4)	0.00	34,956 (62.5)	34,970 (62.5)	0.00
Ischaemic heart disease, %	3,899 (8.7)	3,796 (8.5)	0.01	4,944 (8.8)	5,009 (8.9)	0.00
Stroke, %	511 (1.1)	493 (1.1)	0.00	629 (1.1)	615 (1.1)	0.00
Heart failure, %	918 (2.1)	877 (2.0)	0.01	1,147 (2.0)	1,156 (2.1)	0.00
Peripheral vascular disease, %	1,412 (3.2)	1,400 (3.1)	0.00	1,745 (3.1)	1,748 (3.1)	0.00
Non‐diabetic kidney disease, %	2,365 (5.3)	2,281 (5.1)	0.01	3,224 (5.8)	3,272 (5.8)	0.00
Measures of healthcare utilization						
Previous hospitalization, %	1,775 (4.0)	1,703 (3.8)	0.01	2,199 (3.9)	2,204 (3.9)	0.00
Emergency room visit, %	5,054 (11.3)	5,062 (11.3)	0.00	6,388 (11.4)	6,416 (11.5)	0.00
Endocrinologist visit, %	5,176 (11.6)	5,094 (11.4)	0.01	8,546 (15.3)	8,639 (15.4)	0.00
Number of total medications, mean (SD)	2.3 (2.2)	2.3 (2.2)	0.00	2.5 (2.3)	2.5 (2.3)	0.00

Abbreviations: DPP4i, dipeptidyl peptidase 4 inhibitors; GLP‐1RA, glucagon‐like peptide‐1 receptor agonists; SD, standard deviation; St. diff., standardized difference.

^a^ Measured during the 180‐day period prior to canagliflozin, DPP‐4i or GLP‐1RA initiation.

^b^ Weighted estimates based on 1:4 variable ratio propensity score matching.

### 3.2 TreeScan‐detected signals for potential adverse events

When assessing potential serious incident adverse events based on inpatient or emergency room diagnoses, TreeScan identified signals for a potential increased risk of diabetes ketoacidosis associated with canagliflozin initiation compared with the initiation of a comparator medication in both pairwise cohorts (Table 2). Specifically, signals emerged at the fourth level of the ICD9 hierarchical tree in the canagliflozin vs. DPP4 inhibitor cohort (*p* = .043) and at the fourth and fifth level in the canagliflozin vs. GLP1 agonist cohort (*p* = .0006 and *p* = .032, respectively). A complete list of potential signals is provided in the appendix.

**Table 2:** Signals for potential adverse events based on inpatient or emergency department diagnoses among canagliflozin initiators vs. initiators of other diabetes drugs in two pairwise propensity score‐matched cohorts

Potential adverse event (ICD−9 code)^a^	Treelevel	Canagliflozin vs. DPP4i						Canagliflozin vs. GLP−1RA					
		N events Canagliflozin	N events DPP4i	RR	RD^b^	LLR	*p*	N events Canagliflozin	N events GLP−1RA	RR	RD^b^	LLR	*p*
Diabetes with ketoacidosis (250.1)	4	66	60	2.2	2.4	8.0	.043	92	53	2.3	3.1	13.0	.0006
Diabetes, type 2 with ketoacidosis (250.12)	5	41	32	2.6	1.7	6.6	.230	56	30	2.4	2.0	8.4	.032

Abbreviations: DPP‐4i, dipeptidyl peptidase 4 inhibitors; GLP‐1RA, glucagon‐like peptide‐1 receptor agonists; LLR, log‐likelihood ratio; *p*, *p*‐value; RD, rate difference; RR, rate ratio.

^a^ Based on inpatient or emergency department diagnoses (any position).

^b^ Per 1,000 person years.

When we considered potential adverse events based on any diagnoses, including outpatient diagnoses, TreeScan detected signals compatible with a potential increased risk of female and male genital infections associated with the use of canagliflozin compared with the use of a comparator medication in both cohorts (Table 3). Signals emerged at all investigated levels of the ICD9 hierarchical tree and included specific clinical conditions (eg candidiasis of vulva and vagina, balanoposthitis, vaginitis and vulvovaginitis), as well as aspects pertaining to symptoms, laboratory findings or aspects of care related to genital infections (eg pruritus of genital organs, glycosuria, gynaecological examination).

**Table 3:** Signals for potential adverse events based on any diagnoses among canagliflozin initiators vs. initiators of other diabetes drugs in two pairwise propensity score‐matched cohorts

Potential adverse event (ICD−9 code)^a^	Tree level	Canagliflozin vs. DPP4i						Canagliflozin vs. GLP−1RA					
		N events Canagliflozin	N events DPP4i	RR	RD^b^	LLR	*p*	N events Canagliflozin	N events GLP−1RA	RR	RD^b^	LLR	*p*
Mycoses (110–118)	2	1,861	2,741	1.4	33.4	44.7	.0001	2,217	2,041	1.4	38.6	63.9	.0001
Candidiasis (112.xx)	3	872	635	2.7	37.7	137.8	.0001	1,047	525	2.6	38.3	172.9	.0001
Candidiasis of vulva and vagina (112.1)	4	498	254	3.9	25.2	137.8	.0001	606	232	3.4	25.5	143.1	.0001
Candidiasis of other urogenital sites (112.2)	4	61	34	3.6	3.0	16.8	.0001	60	29	2.7	2.2	8.4	.0681
Candidiasis of unspecified site (112.9)	4	163	86	3.8	8.2	40.8	.0001	198	86	3.0	7.9	41.2	.0001
Balanoposthitis (607.1)	4	88	70	2.5	3.6	15.8	.0001	83	35	3.1	3.3	14.4	.0002
Inflammatory disease of female pelvic organs (614–616)	2	640	544	2.4	25.0	81.8	.0001	785	489	2.1	24.4	106.1	.0001
Inflammatory disease of cervix vagina and vulva (616.x)	3	606	492	2.5	24.5	87.2	.0001	751	422	2.3	25.4	119.6	.0001
Vaginitis and vulvovaginitis (616.1x)	4	519	363	2.9	23.0	98.8	.0001	661	308	2.8	25.3	135.2	.0001
Vaginitis and vulvovaginitis, unspecified (616.10)	5	507	356	2.8	22.4	95.6	.0001	642	299	2.8	24.5	131.6	.0001
Other disorders of female genital tract (617–629)	2	2,099	3,465	1.2	24.9	9.0	.033	–	–	–	–	–	–
Pruritus of genital organs (698.1)	4	64	33	3.9	3.2	16.7	.0001	88	31	3.7	3.8	21.2	.0001
Glycosuria (791.5)	4	92	53	3.5	4.5	26.7	.0001	104	40	3.4	4.4	23.8	.0001
Gynaecological examination (V72.3x)	4	1,785	2,950	1.2	21.1	9.7	.015	–	–	–	–	–	–
Routine gynaecological examination (V72.31)	5	1,782	2,941	1.2	21.2	9.9	.013	–	–	–	–	–	–

Abbreviations: DPP‐4i, dipeptidyl peptidase 4 inhibitors; GLP‐1RA, glucagon‐like peptide‐1 receptor agonists; LLR, log‐likelihood ratio; *p*, *p*‐value; RD, rate difference; RR, rate ratio.

^a^ Based on inpatient, emergency department, or outpatient diagnoses (any position).

^b^ Per 1,000 person years.

No other clinical entities generated signals that were deemed to require further investigation (see Appendix Table A2‐A5 for all TreeScan generated results).

## 4 DISCUSSION

In this large population‐based study of adult patients with T2D, TreeScan consistently identified diabetic ketoacidosis and genital infection as potential adverse events associated with the initiation of canagliflozin compared with the initiation of other T2D medications. These represent known adverse events associated with canagliflozin and provide a proof of principle that TreeScan may help monitor the safety of new medications.

The most common adverse event with canagliflozin, and other SGLT2 inhibitors, is yeast infections of the genitalia. This is based on data from both observational studies and a recent meta‐analysis of clinical trial data.^25^, ^26^ In total, approximately 6% of patients who are started on an SGLT2 inhibitor experience a yeast infection. Another recognized adverse event with canagliflozin and other SGLT2 inhibitors is diabetic ketoacidosis. Based on clinical trial data and observational research, it can affect up to 1% of patients started on an SGLT2 inhibitor.^7^, ^27^, ^28^ Two other potential adverse events with canagliflozin as identified in the CANVAS trial are bone fracture and amputation.^28^ While neither were detected in our current study, our findings are consistent with other observational studies suggesting that these risks are perhaps restricted only to patients at highest risk (eg older adults with significant comorbid conditions).^29^ Because our study primarily included middle aged adults with relatively few comorbid conditions, this may partially account for why neither adverse event was detected by TreeScan.

The ability of TreeScan to identify recognized adverse events of canagliflozin is relevant for other newly approved medications, especially now that approximately 60% of new medications approved by the FDA undergo an expedited pathway based on shorter and smaller clinical trials, some of which are non‐randomized, relative to the non‐expedited approval pathway.^6^, ^30^

The FDA identifies drug safety as its highest priority, and after a drug is approved its safety is primarily monitored through spontaneous reports. Reports can be submitted by healthcare professionals, patients, drug manufacturers and lawyers.^31^, ^32^ Since spontaneous reports are voluntary, the quality is variable and under‐reporting is common.^33^ In 2017, the FDA released ‘Sentinel Initiative: Final Assessment Report’ which outlined how it would modernize the process of post‐market drug safety surveillance, including the implementation of TreeScan and other data mining tools.^34^ Our study provides a framework for how TreeScan might be applied to identify potential adverse events of newly marketed medications. We have identified four important methodologic aspects to using TreeScan which we will discuss individually.

First, an appropriate comparator should be selected. Identifying an appropriate comparator requires expertise in the clinical domain being studied. We identified DPP4 inhibitors and GLP1 agonists as two potential active comparators since both were second‐line medications for T2D at the time of this investigation.^35^ Using two separate active comparators allowed us evaluate the robustness of our results, but there are many clinical scenarios where only one active comparator exists. In our study, regardless of the active comparator used, diabetic ketoacidosis and genital infection were consistently observed associated adverse events of canagliflozin.

Second, a tree of diagnoses is required. Using ICD9 codes is one approach because the data to construct the hierarchical tree are publicly available. How the tree should then be pruned depends on the clinical context. We excluded groups of diagnoses that were unlikely to represent acute drug reactions (eg congenital diagnoses) to limit false signals. Another approach is to include an unpruned tree that includes all diagnoses, but this slightly decreases power to detect effects of exposure. One potential approach is to include an unpruned tree as a sensitivity analysis, but this can lead to spurious findings.

Another important consideration for sensitivity analyses is the level of the tree to be included. Focusing on the 5th level of the tree alone is similar to just focusing on individual diagnostic codes, whereas including the 4th level accounts for related codes which can improve statistical power. For example, there is a 4th level code for diabetic ketoacidosis and then 5th level codes stemming from that on the type of diabetic ketoacidosis. By including the 4th level code, the statistical power to detect diabetic ketoacidosis is improved because grouping at the 4th level accounts for the individual 5th level codes.

Third, studies applying TreeScan thus far have focused on relatively short time horizons of a month or two. We selected a maximum duration of 180 days based on prior literature which identified that the median duration of follow‐up available for adults with diabetes who newly start a diabetes medication within the MarketScan database is approximately 180 days.^7^ Shorter or longer durations can be used depending on the clinical context.

Fourth, multiple testing adjusted *p*‐value are determined through ranking of the test statistics from datasets simulated under the null and the observed dataset from largest to smallest. We ran 9,999 simulations, as fewer simulations would provide less stable ranking. Together with risk estimates, these *p*‐values are used as a means to rank and prioritize alerts for further investigation.

While TreeScan is a powerful data mining tool, it has important limitations. First, the emergence of specific safety signals, that is diabetic ketoacidosis and genital infections, in our study does not necessarily mean canagliflozin is safe with regard to other potential adverse events. Instead, the results need to be interpreted within the context of the healthcare database. For example, we used the MarketScan database which typically includes adults under the age of 65 and thus our results might not generalize well to patients who are older than 65 years of age. Second, potential adverse events of canagliflozin that lacked a specific diagnosis code (eg light headedness) may have not been identified because of limitations with diagnostic codes. Third, intrinsic to all data mining tools, the observed signals require replication, ideally with a focused pharmacoepidemiologic study to evaluate individual signals of interest. This is particularly important since 95% confidence intervals cannot be calculated using TreeScan. Replication of these signals was confirmed by our study team in two such targeted pharmacoepidemiological investigations.^25^

## 5 CONCLUSION

In a large population‐based study, we identified known but no other adverse events associated with canagliflozin, providing reassurance on its safety among adult patients with T2D. The results of our study demonstrate that TreeScan may aid in the process of studying the safety of newly approved medications soon after approval, providing information on signals for potential adverse events in near real time. Additional studies will be necessary to understand the settings where this approach works well and settings where it may not.

## CONFLICTS OF INTEREST

Dr. Patorno is co‐investigator of an investigator‐initiated grant to the Brigham and Women's Hospital from Boehringer‐Ingelheim, not directly related to the topic of the submitted work. Dr. Donald Redelmeier has received funding from a Canada Research Chair in Medical Decision Sciences, the Canadian Institutes of Health Research and the BrightFocus Foundation. Dr. Schneeweiss is consultant to WHISCON, LLC and to Aetion, Inc., a software manufacturer of which he also owns equity. He is principal investigator of investigator‐initiated grants to the Brigham and Women's Hospital from Genentech, Bayer and Boehringer Ingelheim not directly related to the topic of this manuscript. Dr. Kulldorff was supported by National Institute of General Medical Sciences Grant RO1GM108999. Ms. Vine worked for a consulting company where some of her clients were pharmaceutical companies and her projects involved diabetes drugs, but is no longer employed there and none of her projects there relate to this paper. Dr. Wang received salary support from investigator‐initiated grants to the Brigham and Women's Hospital from Boehringer‐Ingelheim, Novartis Pharmaceuticals and Johnson & Johnson, unrelated to this work.

## AUTHOR CONTRIBUTION

All authors involved in study concept and design, acquisition of data, analysis/interpretation of data, critical revision of the manuscript and statistical analysis. Fralick M and Patorno E drafted the manuscript.

## Data Availability

The results that support the findings of this study are available in the supplementary material of this article. The TreeScan algorithm is freely available [https://www.treescan.org/]. The data used in this study are not available.

## 1

**Table A1:** Baseline characteristics prior to matching

	Canagliflozin (*N *= 54540)		DPP4 inhibitor (*N *= 130325)		St. diff
Age (SD)	54.5	(9.6)	59.1	(12.2)	−0.42
Male	28783	52.8%	70038	53.7%	−0.02
Diabetic Nephropathy	2175	4.0%	5933	4.6%	−0.03
Diabetic Retinopathy	2453	4.5%	4938	3.8%	0.04
Diabetic Neuropathy	5340	9.8%	10527	8.1%	0.06
Hypertension	34485	63.2%	81027	62.2%	0.02
Stroke	564	1.0%	3126	2.4%	−0.11
Ischemic Heart Disease	4665	8.6%	16202	12.4%	−0.13
Heart Failure	1039	1.9%	6189	4.7%	−0.16
Non‐Diabetic Renal Disease	2658	4.9%	14588	11.2%	−0.23
Peripheral Vascular Disease	1762	3.2%	4841	3.7%	−0.03
Metformin Use	34723	63.7%	74416	57.1%	0.13
Insulin Use	16523	30.3%	15340	11.8%	0.47
GLP1 Use	10153	18.6%	3458	2.7%	0.54
Endocrinologist	9507	17.4%	7450	5.7%	0.37
Number of Diabetes Medications (SD)	1.2	(0.9)	1.0	(0.8)	0.26
Number of Total Medications (SD)	2.4	(2.1)	2.1	(2.2)	0.15
Emergency room visit	6014	11.0%	18140	13.9%	−0.09
Previous hospitalization	1971	3.6%	10925	8.4%	−0.20

	Canagliflozin (*N *= 70123)		GLP1 Agonist (74820)		St. Diff
Age (SD)	55.1	9.8	54.5	10.3	0.06
Male	38790	55.3%	34020	45.5%	0.20
Diabetic Nephropathy	2597	3.7%	3523	4.7%	−0.05
Diabetic Retinopathy	3001	4.3%	3036	4.1%	0.01
Diabetic Neuropathy	6355	9.1%	7276	9.7%	−0.02
Hypertension	44693	63.7%	45317	60.6%	0.07
Stroke	736	1.0%	878	1.2%	−0.01
Ischemic Heart Disease	6152	8.8%	6824	9.1%	−0.01
Heart Failure	1293	1.8%	1803	2.4%	−0.04
Non‐Diabetic Renal Disease	3456	4.9%	5448	7.3%	−0.10
Peripheral Vascular Disease	2366	3.4%	2195	2.9%	0.03
Metformin Use	38629	55.1%	42971	57.4%	−0.05
Insulin Use	16616	23.7%	21175	28.3%	−0.11
DPP Use	14902	21.3%	10437	13.9%	0.19
Endocrinologist	10041	14.3%	11708	15.6%	−0.04
Number of Diabetes Medications (SD)	1.2	(1.0)	1.1	(0.9)	0.12
Number of Total Medications (SD)	2.5	(2.3)	2.5	(2.3)	0.00
ER Visit	7354	10.5%	9283	12.4%	−0.06
Hospitalizations in previous 180 days	2437	3.5%	3503	4.7%	−0.06

Abbreviations: DPP4i, dipeptidyl peptidase 4 inhibitors; GLP‐1RA, glucagon‐like peptide‐1 receptor agonists; SD, standard deviation; St. diff., standardized difference.

**Table A2:** Signals for potential adverse events based on inpatient or emergency department diagnoses among canagliflozin initiators vs. initiators of DDP4 inhibitors in a propensity score matched cohort

Potential adverse event (ICD−9 code)^a^	Tree level	N events Canagliflozin	N events DPP4i	LLR	*p*
250.1x: Diabetes with ketoacidosis	4	66	60	8.0	.04
250.12: Diabetes with ketoacidosis, type II or unspecified type, uncontrolled	5	41	32	6.6	.23
806.x: Fracture of vertebral column with spinal cord injury	3	4	0	6.0	.43
464.0x: Acute laryngitis	4	7	1	5.4	.64
464.x: Acute laryngitis and tracheitis	3	9	3	4.8	.89
996.66: Infection and inflammatory reaction due to internal joint prosthesis	5	5	0	4.7	.92
620.1: Corpus luteum cyst or hematoma	5	4	0	4.7	.94
620.1x: Corpus luteum cyst or hematoma	4	4	0	4.7	.94
410.3x: Acute myocardial infarction of inferoposterior wall	4	7	2	4.6	.94
110–118: Mycoses	2	66	79	4.5	.96
336.x: Other diseases of spinal cord	3	8	2	4.5	.96
410.31: Acute myocardial infarction of inferoposterior wall, initial episode of care	5	6	1	4.5	.97
782.6x: Pallor and flushing	4	4	0	4.3	.99
464.00: Acute laryngitis without mention of obstruction	5	6	1	4.2	.99
784.4x: Voice disturbance	4	6	1	4.1	.99
112.xx: Candidiasis	3	50	61	4.1	.99
411.0: Postmyocardial infarction syndrome	5	3	0	4.0	.99
411.0x: Postmyocardial infarction syndrome	4	3	0	4.0	.99
402.0x: Malignant hypertensive heart disease	4	6	1	4.0	.99
624.x: Noninflammatory disorders of vulva and perineum	3	4	0	3.9	.99
534.xx: Gastrojejunal ulcer	3	3	0	3.8	.99

Abbreviations: DPP‐4i: dipeptidyl peptidase 4 inhibitors; LLR: log‐likelihood ratio; *p*: *p*‐value.

^a^ Based on inpatient or emergency department diagnoses (any position).

**Table A3:** Signals for potential adverse events based on any diagnoses among canagliflozin initiators vs. initiators of DDP4 inhibitors in a propensity score matched cohort

Potential adverse event (ICD−9 code)^a^	Tree level	N events Canagliflozin	N events DPP4i	LLR	*p*
112.xx: Candidiasis	3	872	635	154.8	.0001
112.1: Candidiasis of vulva and vagina	4	498	254	137.8	.0001
112.1x: Candidiasis of vulva and vagina	5	498	254	137.8	.0001
616.1x: Vaginitis and vulvovaginitis	4	519	363	98.8	.0001
616.10: Vaginitis and vulvovaginitis, unspecified	5	507	356	95.6	.0001
616.x: Inflammatory disease of cervix vagina and vulva	3	606	492	87.2	.0001
614–616: Inflammatory Disease Of Female Pelvic Organs	2	640	544	81.8	.0001
110–118: Mycoses	2	1861	2741	44.7	.0001
112.9: Candidiasis of unspecified site	5	163	86	40.8	.0001
112.9x: Candidiasis of unspecified site	4	163	86	40.8	.0001
791.5: Glycosuria	5	92	53	26.7	.0001
791.5x: Glycosuria	4	92	53	26.7	.0001
112.2: Candidiasis of other urogenital sites	5	61	34	16.8	.0001
112.2x: Candidiasis of other urogenital sites	4	61	34	16.8	.0001
698.1: Pruritus of genital organs	5	64	33	16.7	.0001
698.1x: Pruritus of genital organs	4	64	33	16.7	.0001
607.1: Balanoposthitis	5	88	70	15.8	.0001
607.1x: Balanoposthitis	4	88	70	15.8	.0001
V72.31: Routine gynaecological examination	5	1782	2941	9.9	.013
V72.3x: Gynaecological examination	4	1785	2950	9.7	.015
617–629: Other Disorders Of Female Genital Tract	2	2099	3465	9.0	.033
110.3: Dermatophytosis of groin and perianal area	5	70	75	7.1	.27
110.3x: Dermatophytosis of groin and perianal area	4	70	75	7.1	.27
460–466: Acute Respiratory Infections	2	4892	8723	6.7	.36
783.21: Loss of weight	5	203	309	6.4	.46
627.xx: Menopausal and postmenopausal disorders	3	605	982	6.4	.47
739.xx: Nonallopathic lesions not elsewhere classified	3	1232	2054	6.4	.48
847.0: Sprain of neck	5	225	322	6.3	.53
847.0x: Sprain of neck	4	225	322	6.3	.53
783.2x: Abnormal loss of weight and underweight	4	203	311	6.2	.58
605.x: Redundant prepuce and phimosis	4	46	39	6.1	.64
605.x: Redundant prepuce and phimosis	3	46	39	6.1	.64
605: Redundant prepuce and phimosis	5	46	39	6.1	.64
623.5: Leucorrhoea, not specified as infective	5	105	112	6.0	.66
623.5x: Leucorrhoea, not specified as infective	4	105	112	6.0	.66
623.x: Noninflammatory disorders of vagina	3	159	195	5.9	.69
847.x: Sprains and strains of other and unspecified parts of back	3	585	941	5.3	.92
577.x: Diseases of pancreas	3	253	393	5.2	.93
410.31: Acute myocardial infarction of inferoposterior wall, initial episode of care	5	7	1	5.0	.96
487.1: Influenza with other respiratory manifestations	5	234	344	4.9	.98
487.1x: Influenza with other respiratory manifestations	4	234	344	4.9	.98
696.1x: Other psoriasis	4	171	236	4.7	.99
696.1: Other psoriasis	5	171	236	4.7	.99
900.xx: Injury to blood vessels of head and neck	3	4	0	4.7	.99
577.1x: Other psoriasis	4	41	43	4.7	.99
577.1: Other psoriasis	5	41	43	4.7	.99
373.12: Hordeolum internum	5	47	53	4.6	.99
461.x: Acute sinusitis	3	1674	2870	4.6	.99
526.4x: Inflammatory conditions of jaw	4	10	4	4.6	.99
526.4: Inflammatory conditions of jaw	5	10	4	4.6	.99
461.9x: Acute sinusitis, unspecified	4	1226	2100	4.4	.99
461.9: Acute sinusitis, unspecified	5	1226	2100	4.4	.99
V76.4x: Special screening for malignant neoplasms of other sites	4	763	1322	4.3	.99
526.xx: Diseases of the jaws	3	24	22	4.2	.99
900.9x: Injury to unspecified blood vessel of head and neck	4	3	0	4.2	.99
900.9: Injury to unspecified blood vessel of head and neck	5	3	0	4.2	.99
470–478: Other Diseases Of Upper Respiratory Tract	2	2788	4992	4.1	.99
730.37: Periostitis, without mention of osteomyelitis, ankle and foot	5	8	2	4.1	.99
686.1: Pyogenic granuloma of skin and subcutaneous tissue	5	27	23	4.1	.99
686.1x: Pyogenic granuloma of skin and subcutaneous tissue	4	27	23	4.1	.99
487.x: Influenza	3	253	386	4.1	.99
V88.02: Acquired absence of uterus with remaining cervical stump	5	3	0	4.0	.99
598.0: Urethral stricture due to infection	4	3	0	4.0	.99
349.9x: Unspecified disorders of nervous system	4	15	9	4.0	.99
349.9: Unspecified disorders of nervous system	5	15	9	4.0	.99

Abbreviations: DPP‐4i: dipeptidyl peptidase 4 inhibitors; LLR: log‐likelihood ratio; *p*: *p*‐value.

^a^ Based on inpatient, emergency department or outpatient diagnoses (any position).

**Table A4:** Signals for potential adverse events based on inpatient or emergency department diagnoses among canagliflozin initiators vs. initiators of GLP1 agonists in a propensity score matched cohort

Potential adverse event (ICD−9 code)^a^	Tree level	N events Canagliflozin	N events GLP−1RA	LLR	*p*
250.1x: Diabetes with ketoacidosis	4	92	53	13.0	.0006
250.12: Diabetes, type 2 with ketoacidosis	5	56	30	8.4	.032
574.50: Calculus of bile duct without mention of cholecystitis, without mention of obstruction	5	13	2	6.2	.33
112.x: Candidiasis	3	59	42	6.0	.39
110–118: Mycoses	2	74	58	5.4	.65
278.1: Localized adiposity	5	5	0	5.2	.71
278.1x: Localized adiposity	4	5	0	5.2	.71
112.1x: Candidiasis of vulva and vagina	4	19	7	5.1	.76
112.1: Candidiasis of vulva and vagina	5	19	7	5.1	.76
574.3x: Calculus of bile duct with acute cholecystitis	4	6	0	4.9	.85
967.xx: Poisoning by sedatives and hypnotics	3	6	0	4.9	.85
788.1x: Dysuria	4	40	26	4.8	.89
788.1: Dysuria	5	40	26	4.8	.89
969.x: Poisoning by psychotropic agents	3	10	4	4.5	.95
426.10: Atrioventricular block, unspecified	5	5	0	4.4	.97
847.9: Sprain of unspecified site of back	5	33	20	4.3	.98
847.9x: Sprain of unspecified site of back	4	33	20	4.3	.98
574.30: Calculus of bile duct with acute cholecystitis, without mention of obstruction	5	5	0	4.2	.99
443.0x: Raynaud's syndrome	4	4	0	4.2	.99
443.0: Raynaud's syndrome	5	4	0	4.2	.99
V70.x: General medical examination	3	17	7	4.1	.99
799.89: Other ill‐defined conditions	5	16	7	3.9	.99
799.8x: Other ill‐defined conditions	4	16	7	3.9	.99
V68.89: Encounters for other specified administrative purpose	5	3	0	3.7	.99
V68.8x: Encounters for other specified administrative purpose	4	3	0	3.7	.99

Abbreviations: GLP‐1RA: glucagon‐like peptide‐1 receptor agonists; LLR: log‐likelihood ratio; *p*: *p*‐value.

^a^ Based on inpatient or emergency department diagnoses (any position).

**Table A5:** Signals for potential adverse events based on any diagnoses among canagliflozin initiators vs. initiators of GLP1 agonists in a propensity score matched cohort

Potential adverse event (ICD−9 code)^a^	Tree level	N events Canagliflozin	N events GLP−1RA	LLR	*p*
112.xx: Candidiasis	3	1047	525	172.9	.0001
112.1: Candidiasis of vulva and vagina	4	606	232	143.1	.0001
112.1x: Candidiasis of vulva and vagina	5	606	232	143.1	.0001
616.1x: Vaginitis and vulvovaginitis	4	661	308	135.2	.0001
616.10: Vaginitis and vulvovaginitis, unspecified	5	642	299	131.6	.0001
616.x: Inflammatory disease of cervix vagina and vulva	3	751	422	119.6	.0001
614–616: Inflammatory Disease Of Female Pelvic Organs	2	785	489	106.1	.0001
110–118: Mycoses	2	2217	2041	63.9	.0001
112.9: Candidiasis of unspecified site	4	198	86	41.2	.0001
112.9x: Candidiasis of unspecified site	5	198	86	41.2	.0001
791.5: Glycosuria	4	104	40	23.8	.0001
791.5x: Glycosuria	5	104	40	23.8	.0001
698.1x: Pruritus of genital organs	4	88	31	21.2	.0001
698.1: Pruritus of genital organs	5	88	31	21.2	.0001
607.1x: Balanoposthitis	4	83	35	14.4	.0002
607.1: Balanoposthitis	5	83	35	14.4	.0002
112.2x: Candidiasis of other urogenital sites	4	60	29	8.4	.068
112.2: Candidiasis of other urogenital sites	5	60	29	8.4	.068
250.12: Diabetes, type 2 with ketoacidosis	5	62	36	8.4	.071
623.5x: Leucorrhoea, not specified as infective	4	131	105	8.0	.10
623.5: Leucorrhoea, not specified as infective	5	131	105	8.0	.10
698.x: Pruritus and related conditions	3	352	343	7.4	.20
V72.31: Routine gynaecological examination	5	2293	2929	7.4	.21
V72.3x: Gynaecological examination	4	2295	2935	7.2	.25
458.x: Hypotension	3	297	302	6.9	.32
372.30: Conjunctivitis, unspecified	5	199	186	6.8	.36
117.9x: Other and unspecified mycoses	4	33	18	6.6	.41
117.9: Other and unspecified mycoses	5	33	18	6.6	.41
307.46: Sleep arousal disorder	5	8	0	6.1	.66
577.x: Diseases of pancreas	3	273	280	5.9	.74
372.3x: Diseases of pancreas	4	218	212	5.8	.74
250.1x: Diabetes with ketoacidosis	4	120	105	5.6	.81
788.4x: Frequency of urination and polyuria	4	621	678	5.4	.88
458.0x: Orthostatic hypotension	4	90	74	5.1	.96
458.0: Orthostatic hypotension	5	90	74	5.1	.96
577.1: Chronic pancreatitis	5	49	32	5.0	.97
577.1x: Chronic pancreatitis	4	49	32	5.0	.97
736.72: Equinus deformity of foot, acquired	5	53	34	4.8	.99
727.02: Giant cell tumour of tendon sheath	5	6	0	4.7	.99
V86: Oestrogen Receptor Status	2	24	10	4.6	.99
V86.xx: Oestrogen receptor status	3	24	10	4.6	.99
070: Viral hepatitis	5	6	0	4.5	.99
070.x: Viral hepatitis	4	6	0	4.5	.99
117.x: Other mycoses	3	33	22	4.4	.99
783.2x: Abnormal loss of weight and underweight	4	239	239	4.4	.99
783.21: Loss of weight	5	239	239	4.4	.99
V70.1x: General psychiatric examination, requested by the authority	4	4	0	4.3	.99
V70.1: General psychiatric examination, requested by the authority	5	4	0	4.3	.99

Abbreviations: GLP‐1RA: glucagon‐like peptide‐1 receptor agonists; LLR: log‐likelihood ratio; *p*: *p*‐value.

^a^ Based on inpatient, emergency department, or outpatient diagnoses (any position).

Additional details on follow up time.

For GLP, exposed mean time is 134 days (standard deviation [SD] = 53). Unexposed mean time is 132 days (SD = 53). Unexposed weighted mean time is 130 days (SD = 47). Overall unweighted mean time is 134 days (SD=54), and overall weighted mean time is 134 days (SD 50).

For DPP exposed mean time is 136 days (SD = 54), unexposed mean time is 135 days (SD = 53), and unexposed weighted mean time is 132 days (36). Overall unweighted mean time is 135 days (54), and overall weighted mean time is 134 days (43).
